# Recent Development of Genetic Code Expansion for Posttranslational Modification Studies

**DOI:** 10.3390/molecules23071662

**Published:** 2018-07-08

**Authors:** Hao Chen, Sumana Venkat, Paige McGuire, Qinglei Gan, Chenguang Fan

**Affiliations:** 1 Cell and Molecular Biology Program, University of Arkansas, Fayetteville, AR 72701, USA; hc019@uark.edu (H.C.); sv009@uark.edu (S.V.); 2 Department of Biological Sciences, University of Arkansas, Fayetteville, AR 72701, USA; plmcguir@uark.edu; 3 Department of Chemistry and Biochemistry, University of Arkansas, Fayetteville, AR 72701, USA; qingleig@uark.edu

**Keywords:** genetic code expansion, noncanonical amino acid, unnatural amino acid, posttranslational modification, protein acetylation, protein phosphorylation, protein methylation, protein oxidation

## Abstract

Nowadays advanced mass spectrometry techniques make the identification of protein posttranslational modifications (PTMs) much easier than ever before. A series of proteomic studies have demonstrated that large numbers of proteins in cells are modified by phosphorylation, acetylation and many other types of PTMs. However, only limited studies have been performed to validate or characterize those identified modification targets, mostly because PTMs are very dynamic, undergoing large changes in different growth stages or conditions. To overcome this issue, the genetic code expansion strategy has been introduced into PTM studies to genetically incorporate modified amino acids directly into desired positions of target proteins. Without using modifying enzymes, the genetic code expansion strategy could generate homogeneously modified proteins, thus providing powerful tools for PTM studies. In this review, we summarized recent development of genetic code expansion in PTM studies for research groups in this field.

## 1. Introduction

Commonly, besides 3 stop codons, the genetic code of life contains 61 triplet codons which can encode 20 canonical amino acids. Although these amino acids are the basic composition of natural proteins, many proteins still need additional modifications of amino acid residues to be properly functional. For this purpose, cells utilize posttranslational modifications (PTMs) such as phosphorylation, acetylation, methylation and ubiquitination to modulate the activity, localization and other properties of a protein [1,2], thus regulating a variety of biological processes such as gene transcription, protein biosynthesis, cellular signaling and metabolism [3,4,5].

There are several challenges to study PTMs. Firstly, PTMs are very dynamic in cells and most of them are reversible, so it is difficult to separate a purely modified protein. Secondly, PTMs happen at multiple sites simultaneously in a single protein and various PTMs could compete with the same amino acid residue, making the characterization of one particular PTM at one specific site difficult. To do so, the most rigorous approach is to insert the modified amino acid directly at the desired position in proteins. Obviously, co-translationally (rather than posttranslationally) incorporating modified amino acids into proteins is one of best ways. In 1956, selenomethionine was first demonstrated to be incorporated into proteins at methionine residues in bacterial cells [6] and then lots of amino acid analogs, which could be substrates for the natural translational machinery, were identified to replace their natural counterparts in proteins [7]. Besides this residue-specific strategy, several approaches have been developed to incorporate noncanonical amino acids (ncAAs) into a protein site-specifically. Among them, the genetic code expansion strategy is the most popular one [8,9,10].

**Figure 1:**
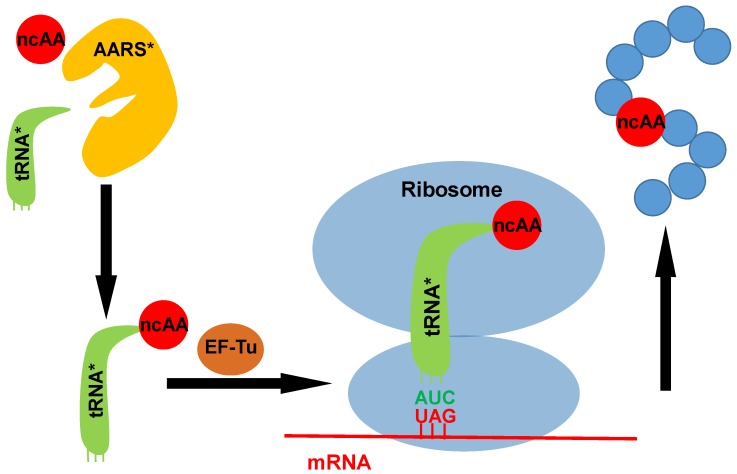
A scheme for the genetic code expansion strategy. The introduced orthogonal aminoacyl-tRNA synthetase (AARS) charges its cognate tRNA with one ncAA. Then the ncAA-charged tRNA is brought to the ribosome by EF-Tu. The introduced tRNA with a designed anticodon can read the corresponding codon in the mRNA (UAG is probably the most reassigned codon used for co-translational incorporation of posttranslational modifications (PTMs).), then direct the incorporation of ncAA into the specific site of the target protein. AARS*: introduced aminoacyl-tRNA synthetase; tRNA*: introduced tRNA; ncAA: noncanonical amino acid.

Typically, a pair of an aminoacyl-tRNA synthetase (AARS) and its cognate tRNA from different domains of life is introduced into host cells. Such pairs of AARS/tRNA are called orthogonal pairs, as they usually do not cross-react with endogenous pairs of AARS/tRNA in host cells. The introduced AARS or its engineered variant charges the orthogonal tRNA with a specifically recognized ncAA. Then the ncAA-charged tRNA is escorted by the elongation factor-Tu (EF-Tu) to the ribosome where it reads an assigned codon (commonly a stop codon) on the mRNA and directs the incorporation of the ncAA into the protein at the assigned position (Figure 1) [11]. Currently, the most widely used orthogonal AARS/tRNA pairs to achieve ncAA incorporation are derived from either the pair of pyrrolysyl-tRNA synthetase (PylRS)/tRNA^Pyl^ from *Methanosarcina* species [12,13] or the pair of tyrosyl-tRNA synthetase (TyrRS)/tRNA^Tyr^ from *Methanococcus jannaschii* [14]. Since the selected codon for ncAA incorporation could be substituted for the original codon in the target gene by site-directed mutagenesis, the genetic code expansion strategy could be employed to incorporate ncAAs into specific positions of target proteins in living cells, thus providing powerful tools for biological studies such as labelling proteins for microscopic and proteomic studies; encoding photo-crosslinkers for mapping weak, transient and pH-sensitive protein interactions; incorporating photo-caged amino acids for controlling reactions by light; and introducing biophysical probes and labels for monitoring proteins [15,16,17,18,19,20,21]. In this review, we focus on its application in PTM studies.

## 2. Lysine Acetylation and its Analogs by Genetic Code Expansion 

Lysine acetylation, which was firstly discovered in histone, targets the ε-amino group of lysine residues [22,23]. It is a reversible process catalyzed by lysine acetyltransferases and deacetylases, which interact with each other to regulate acetylation levels of proteins in cells [24,25,26]. As a well-studied example, histone acetylation plays a crucial role in regulating gene transcription [27,28]. On the other hand, non-histone acetylation has also been proved to be important in multiple cellular processes such as gene expression, metabolic regulation and cell signaling [29,30]. 

To study lysine acetylation, several genetic incorporation systems for acetyllysine (AcK) have been developed. In 2008, Neumann et al. firstly demonstrated the site-specific incorporation of AcK in recombinant proteins produced in *Escherichia coli* (*E. coli*) cells by engineering the PylRS to recognize AcK specifically [31]. Later, this AcK-incorporation system was successfully introduced into mammalian cells by Mukai et al. [32]. By a different engineering strategy, Umehara et al. obtained another AcK-specific PylRS variant (AcKRS) with a different binding pocket for AcK [33]. However, all these AcKRS variants had dramatic decrease in catalytic efficiency during engineering [18]. To optimize AcK-incorporation systems, several approaches have been performed. Huang et al. enhanced amber suppression by overexpressing the C-terminal domain of the ribosomal protein L11 to decrease release factor 1-mediated termination of protein translation and they were able to incorporate three AcK residues into one protein simultaneously [34]. Fan et al. further engineered tRNA^Pyl^ for better binding with EF-Tu, increasing AcK-incorporation efficiency by 3-fold in *E. coli* cells [35]. Recently, Bryson et al. utilized the phage-assisted continuous evolution to evolve PylRS over its full sequence rather than the amino acid binding site alone and the resulting AcKRS variant had a 10-fold increase in the incorporation efficiency [36]. Later, our group combined the optimized AcKRS variant and tRNA^Pyl^ mutant to establish a facile protocol for AcK incorporation, allowing the read-through of the amber stop codon up to 70% [37]. Till now, genetic incorporation of AcK has been successful in bacteria [31,38], yeast [39], mammalian cells [32,40,41] and animals [42]. 

**Figure 2:**
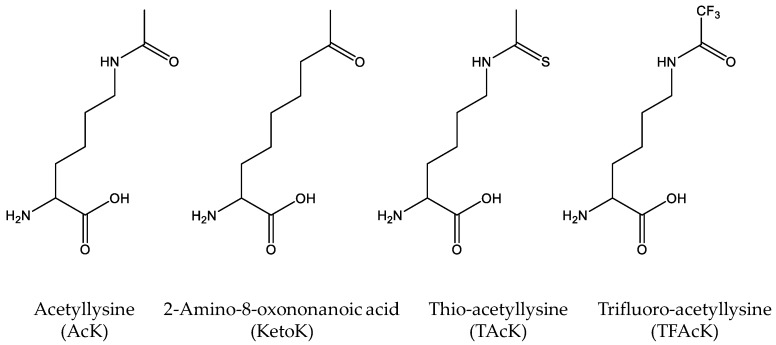
The Structures of acetyllysine (AcK) and its non-deacetylatable analogs, including 2-amino-8-oxononanoic acid (KetoK), thio-acetyllysine (TAcK) and trifluoro-acetyllysine (TFAcK).

As mentioned, cells have different kinds of deacetylases to remove acetyl-groups from the acetylated lysine residues. Although deacetylase inhibitors such as nicotinamine are commonly added into growth media for overexpressing site-specifically acetylated proteins, cells may still have residual deacetylase activities, making it possible that genetically-incorporated acetylated lysine residues could be deacetylated during expression and purification. To solve this potential problem, non-deacetylatable AcK analogs are needed (Figure 2). Huang et al. designed a nonhydrolyzable 2-amino-8-oxononanoic acid (KetoK) and genetically incorporated it into proteins [43]. Later, by flexizyme-mediated tRNA aminoacylation, Xiong et al. were able to incorporate thio-acetyllysine (TAcK) into histone H3 site-specifically with the cell-free translation system [44]. Recently, our group further engineered the AcKRS for recognition of TAcK and successfully incorporated TAcK into proteins in *E. coli* cells [45]. We showed that TAcK residues could be recognized by the AcK antibody and the effect of thioacetylation was similar to that of acetylation on the enzyme activities of malate dehydrogenase, indicating that TAcK could be an ideal mimic of AcK in acetylation studies. Furthermore, we confirmed that TAcK residues could resist the deacetylase [45]. This system will be particularly useful if long-lasting effects of acetylation need to be determined in living cells with the concern of endogenous deacetylases. Very recently, Zhang et al. genetically incorporated trifluoro-acetyllysine (TFAcK) into p53 to detect the conformational changes by NMR (nuclear magnetic resonance). They also demonstrated that the TFAcK-containing p53 protein could not be deacetylated by sirtuin deacetylase [46]. 

The genetic code expansion strategy has been widely used to study the lysine acetylation. Due to the importance of histone acetylation in gene transcription, Neumann et al. genetically incorporated AcK into core histones including H2A, H2B and H3 [47], however it was hard to get recombinant H4 with acetylation. Mukai et al. and Wakamori et al. used the cell-free translation system to incorporate AcK into H4 at four positions [48,49]. Recently, Wilkins et al. improved AcK incorporation into H4 by constructing a gene fusion coding for H3 connected to H4 by a proper linker [50]. There were also many studies on non-histone protein acetylation by this technique. For instance, Pan et al. used the genetic code expansion strategy to incorporate AcK into human peroxiredoxin 1 (hPrX1) at position K27 and found that the acetylation of hPrx1 changed its biological role in different environments [51]. As another example, de Boor et al. genetically incorporated AcK into GTP-binding protein Ran to demonstrate its role in nucleotide exchange and hydrolysis, as well as the interaction with import and export receptors [52]. Recently, Ohtake et al. has applied genetic code expansion to show that ubiquitin acetylation inhibits polyubiquitin chain elongation [53]. Besides eukaryotes, more and more proteomic studies have shown that lysine acetylation is also widely distributed in prokaryotic cells, enriched in metabolic pathways and protein biosynthesis [54]. Recently, our group has applied the genetic code expansion in studying two metabolic enzymes, malate dehydrogenase (MDH) and isocitrate dehydrogenase (ICDH) of *E. coli* [55,56]. Interestingly, although both enzymes are in the tricarboxylic acid (TCA) cycle, the effects of lysine acetylation are completely different: acetylation of MDH increased the enzyme activity, while acetylation of ICDH decreased the enzyme activity. We also characterized lysine acetylation of *E. coli* TyrRS and showed that the acetylation impaired the TyrRS activity by neutralizing positive charges of lysine residues binding to ATP, the substrate for tRNA aminoacylation [57]. 

## 3. Lysine Ubiquitination by Combining Genetic Code Expansion with Native Chemical Ligation

**Figure 3:**
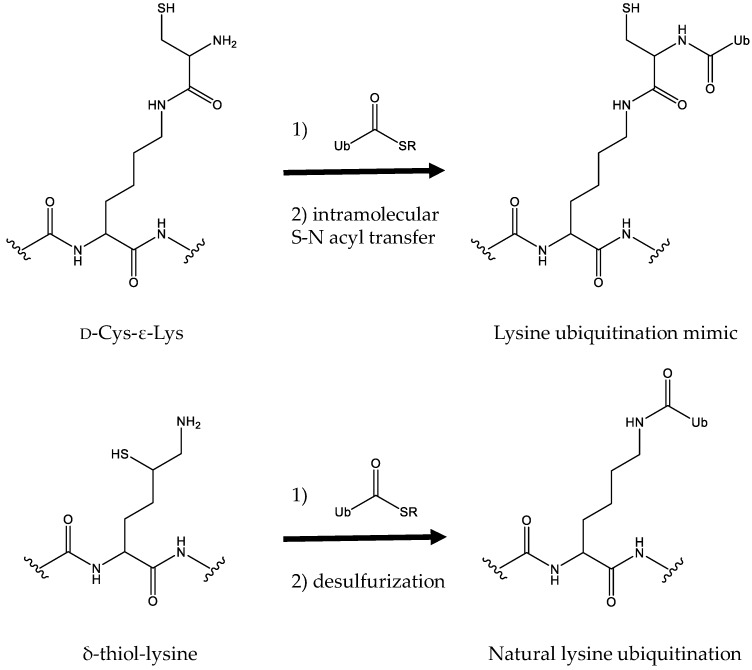
The schemes for producing ubiquitinated proteins. In both approaches, a precursor is genetically incorporated into proteins first, which was linked to a modified ubiquitin harboring a C-terminal thioester by native chemical ligation and then formed ubiquitinated proteins by rearrangement or group removal.

Different from lysine acetylation which modifies lysine residues with a small acetyl-group, lysine ubiquitination covalently attaches the carboxyl terminus of a 76-amino acid protein, ubiquitin, to lysine residues [58]. It affects a variety of biological processes, among which the most remarkable function is its role in protein degradation [59]. Several groups have used the native chemical ligation strategy to create ubiquitin conjugates [60,61]. But these approaches need multi-step organic synthesis, which may not be easily performed in a biological laboratory. Later, an easier approach to combine the genetic code expansion strategy and native chemical ligation was developed (Figure 3). Li et al. firstly incorporated d-Cys-ε-Lys into a protein at a selected position, which was reacted with a modified ubiquitin harboring a C-terminal thioester by thiol exchange and then irreversible intramolecular S-N acyl transfer to form a semisynthetic ubiquitinated protein [62]. However, this method could not form the natural isopeptide linkage between proteins and ubiquitin. For this purpose, Virdee et al. engineered PylRS to recognize and incorporate δ-thiol-lysine into proteins and combined it with native chemical ligation and desulfurization to link ubiquitin and substrate proteins with an entirely native isopeptide bond [63] (Figure 3).

## 4. Lysine Methylation by Combining Genetic Code Expansion and Different Chemical Reactions

Lysine methylation, another important lysine modification, was also firstly discovered in histones [23]. Different from other lysine modifications, there are three forms of lysine methylation, including mono-, di- and tri-methylation [64,65]. Unlike histone acetylation, which is often related to transcription activation, histone methylation is involved in both transcription activation and silencing, depending on methylation sites and forms [66,67]. On the other hand, lysine methylation of non-histone proteins also plays important roles in many cellular processes. For example, methyltransferase Set 9 can specifically methylate lysine residues in p53 to regulate its target gene expression [68]. In prokaryotic cells, lysine methylation has been shown to modulate cell motility [69]. Previously, the synthesis of lysine-methylated proteins mainly depended on native chemical ligation [70]. Later, genetic code expansion was introduced into this field but it has been challenging to evolve an AARS to specifically recognize methylated lysine but not lysine itself. Thus, direct incorporation of lysine methylation into protein has not yet been successful. However, several groups have developed alternative methods to genetically incorporate lysine methylation precursors first and then obtain methylated proteins by different physical or chemical reactions [71]. 

For mono-methylation, Nguyen et al. introduced the tert-butyloxycarbonyl (Boc)-methyllysine, which could be removed by acid [72]; Groff et al. and Wang et al. independently incorporated photocaged *N*^ε^-(*o*-nitrobenzylcarbamoyl)-methyllysine, followed by UV exposure to remove the protecting group [73,74]; Ai et al. designed another protected methyllysine, *N*^ε^-allylcarbamoyl-methyllysine, which could be deprotected by chloro-pentamethylcyclopentadienyl-cyclooctadieneruthenium (II) [75]. Yanagisawa et al. used the release factor 1-knocked strain in both cell-based and cell-free systems to incorporate mono-methyllysine into H3 at multiple positions simultaneously [76].

For di-methylation, Nguyen et al. used the Boc group to protect the lysine residue and the benzyloxycarbonyl group to protect all other lysine residues in the target protein. Then the Boc-lysine was deprotected by acid and methylated by reductive alkylation using formaldehyde and a dimethylamine borane complex [77]. Recently, Wang et al. developed a method that could be performed in protein-friendly conditions. They genetically incorporated *N*^ɛ^-(4-azidobenzoxycarbonyl)-δ, ɛ-dehydrolysine into proteins, which was followed by Staudinger reduction with tris-(2-carboxyethyl)phosphine to form allysine and reductive amination with dimethylamine in the presence of NaCNBH_3_ [78]. 

**Figure 4:**
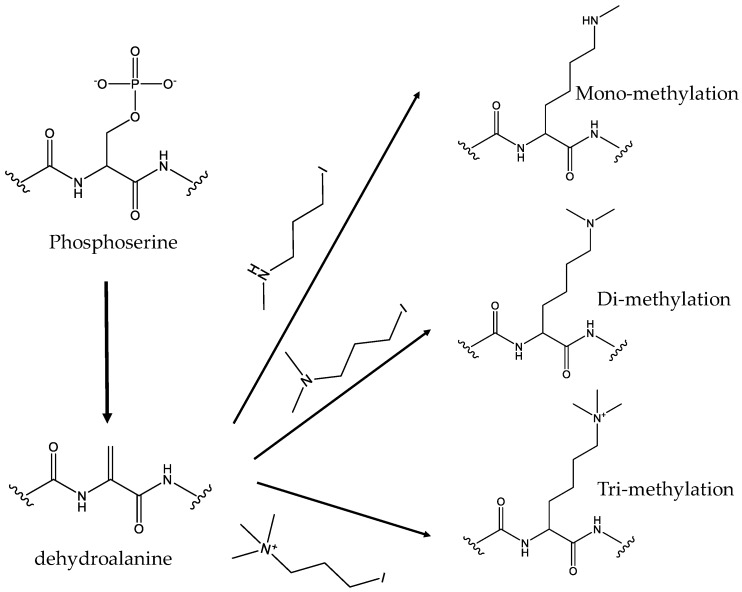
The schemes for producing methylated proteins. Phosphoserine is first site-specifically incorporated into proteins and dephosphorylated to form dehydroalanine, then formed different methylated proteins with different alkyl iodides.

For tri-methylation, Yang et al. utilized the established phosphoserine (Sep) incorporation system to generate Sep-containing proteins first (For more details of Sep-incorporation, please refer to the ‘Serine phosphorylation and its analogs’ section below). Then the Sep residue was dephosphorylated to form dehydroalanine, which was followed by Zn-Cu promoted conjugate addition of 3-iodo-*N,N,N,*-trimethylpropan-1-amine to form tri-methylated proteins [79]. Moreover, by using different alkyl iodides, this approach could also generate monomethyl-, dimethyl-, formyl-, or acetyl-lysine residue at desired sites in proteins, making it an easy route to produce site-specific authentic protein modifications (Figure 4).

## 5. Other Lysine Modifications by Genetic Code Expansion 

**Figure 5:**
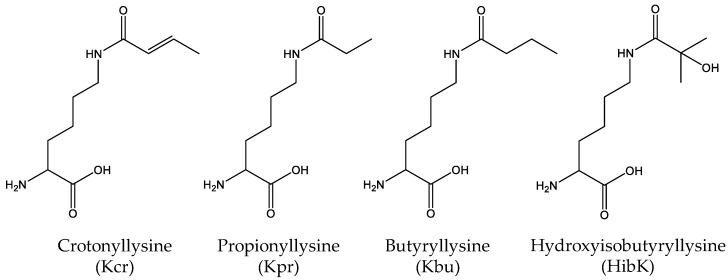
The structures of other modifications of lysine, including crotonylation, propionylation, butyrylation and 2-hydroxyisobutyrylation.

A number of proteomic studies have shown the existence of many other modifications of lysine residues, including crotonylation [80], propionylation [81], butyrylation [81] and 2-hydroxyisobutyrylation [82] (Figure 5). Kim et al. synthesized *N*^ε^-crotonyllysine (Kcr) and used an evolved PylRS variant to efficiently incorporate Kcr into desired positions of recombinant proteins. They successfully applied this system to express a site-specifically crotonylated human histone H2B in *E.coli* and mammalian cells with high fidelity and efficiency [83]. Later, Gattner et al. showed that the wild-type PylRS could be used to incorporate *N*^ε^-propionyl-(Kpr), *N*^ε^-butyryl-(Kbu) and *N*^ε^-crotonyl-lysine (Kcr) into histone H3 [84]. Similarly, Wilkins et al. extended their studies on lysine acetylation of histone H4 to lysine propionylation, butyrylation and crotonylation. This method was then utilized to test the ability of antibodies to distinguish between different lysine modifications in histone H4 [50]. Moreover, Xiao et al. reported a new evolved PylRS variant to site-specifically incorporate *N*^ε^-2-hydroxyisobutyryl-lysine (HibK), a new type of histone mark [85]. Recently, Owens et al. reported a versatile two-tier screen (a white/blue colony screen and a plate-based colorimetric assay) platform to evolve AARSs for better ncAA incorporation and successfully applied this platform to synthesize different forms of lysine acylation [86]. By a different strategy, Wang et al. developed a versatile approach for generating proteins with lysine acylation by combining genetically-encoded azidonorleucine with traceless Staudinger ligation [87]. 

## 6. Arginine Methylation by Genetic Code Expansion

Arginine is another positively charged amino acid and it can be posttranslationally modified with one or two methyl groups [88]. Although Arginine methylation is not as abundant as lysine methylation, it still has important effects on many cellular processes, including RNA processing, transcriptional regulation, signal transduction and DNA repair [89]. By using the in vitro translation system, Akahoshi et al. used the yeast arginyl-tRNA synthetase to charge a yeast tRNA^Arg^-derived tRNA mutant which has a CCCG four-base anticodon with monomethyl-arginine by an EF-Tu variant E215A with an improved aminoacylation efficiency and site-specifically incorporated it into H3 protein at position R8, R17 and R26, demonstrating that R8, R17 methylation could suppress K9 acetylation [90]. In this study, arginine analogs were introduced into proteins without the need for organic synthesis and it will be useful for analyzing the functional roles of arginine modifications in various proteins.

## 7. Serine Phosphorylation and its Analogs by Genetic Code Expansion

As the most abundant PTMs in nature, protein phosphorylation can be found in organisms from all three kingdoms [91,92]. Traditionally, it was believed that bacteria only possess aspartate and histidine phosphorylation which plays roles in sensing the environment and regulating the import of nutrients [93,94] and the phosphorylation of serine, threonine and tyrosine residues was exclusively found in eukaryotes [95]. However, the recent genomic sequencing and proteomic studies demonstrated that the phosphorylation of serine, threonine and tyrosine residues is also distributed widely in prokaryotes and involved in many physiological processes just like that in eukaryotic cells including cell cycle, cell differentiation, cell division, cell metabolism, stress response, as well as protein synthesis [96,97]. 

Due to the importance of phosphorylation, many scientists have tried to develop genetic incorporation systems for phosphorylated amino acids. In 2011, Park et al. made a breakthrough with phosphoserine (Sep) incorporation. It was based on their previous discovery that certain methanogenic archaea used an unusual AARS, phosphoseryl-tRNA synthetase (SepRS), to catalyze the formation of Sep-tRNA^Cys^ for cysteine biosynthesis [98]. Combined with an engineered EF-Tu variant which could bind with negatively-charged amino acids much better than the wild-type EF-Tu, they were able to site-specifically incorporate Sep into proteins [99]. However, the amber codon UAG selected as the signal for Sep incorporation in this system caused low protein yields, since the UAG codon initially is a stop codon to bind with the release factor 1 (RF1) to stop translation. To solve this problem, Heinemann et al. selected a partially recoded RF-1 knockout strain of *E. coli* (EcAR7.ΔA) in which seven original UAG codons of essential genes were converted to UAA codons. After evaluating this improved system by incorporation of Sep into green fluorescent protein (GFP) and serine/threonine-protein kinase WNK4, they suggested that the efficiency of phosphoserine in this system was enhanced but with deleterious effects on cell fitness and viability [100]. Later, by a new proteomic workflow, which can quantify both canonical and non-canonical amino acids in recombinant proteins, they found that although Sep incorporation at UAG codons was enhanced in EcAR7.ΔA, there were still an unexpectedly large number of canonical amino acids incorporated at the UAG codons [101]. To further improve the Sep-incorporation system, Pirman et al. introduced the C321.ΔA strain developed by the Church group (all 321 TAG codons in the *E. coli* genome were substituted with TAA) [102], which not only eliminated all negative effects on cell growth brought by RF1-knockout but also avoided the canonical amino acid incorporation [103]. A similar strategy was also successfully performed in the cell-free translation system to produce multiply-phosphorylated proteins by Oza et al. [104]. Without using genomically modified strains, Lee et al. were able to increase Sep-incorporation efficiency in the commonly used expression strain BL21(DE3) by adopting improved SepRS and EF-Tu variants [105]. Recently, George et al. compared both approaches in their studies on ubiquitin phosphorylation [106]. Interestingly, they found that in the *E. coli* ΔRF1 strain, the UAG codons can be skipped or bypassed by the ribosome to yield proteins with a single residue deleted. 

**Figure 6:**
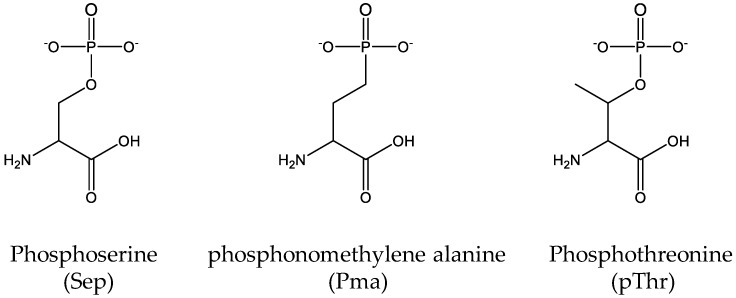
The structures of phosphoserine (Sep), its nonhydrolyzable analog phosphonomethylene alanine (Pma) and phosphothreonine (pThr).

Similar to lysine acetylation studies, it is useful to genetically incorporate nonhydrolyzable analogs of phosphorylated amino acids when endogenous kinases and phosphatases may impede proposed studies. For this purpose, Rogerson et al. genetically incorporated one non-hydrolysable analog of Sep, phosphonomethylene alanine (Pma) (Figure 6) into proteins by their optimized SepRS/tRNA^sep^ pair in a metabolically engineered *E.coli* strain in which the intracellular levels of Sep was decreased by overexpression of *serB* (the phosphoserine phosphatase to remove Sep) and deletion of *serC* (the phosphoserine aminotransferase to produce Sep) from the genome [107]. Recently, our group successfully transferred the Sep-incorporation system into *Salmonella* to produce phosphorylated protein in living cells of *Salmonella* [38]. We demonstrated that the phosphorylation of malate dehydrogenase in *Salmonella* could inhibit the enzyme activity, which played an opposite effect of acetylation of the same enzyme, suggesting that two PTMs could cross-react with each other to regulate enzymes for adapting to different environments. Till now, Sep-incorporation has been successful in *E. coli*, *Salmonella* and mammalian cells [38,99,108]. 

## 8. Threonine Phosphorylation by Genetic Code Expansion

Due to the similarity between phosphothreonine (pThr) and Sep (Figure 6), evolving the SepRS/tRNA^Sep^ pair for the site-specific incorporation of pThr into proteins is feasible. But there are still some challenges. Firstly, different from Sep which exists in cells as the precursor for serine, there is no pThr biosynthesis pathway in *E. coli* cells. And the cellular uptake of negatively charged compounds is relatively low, which cannot provide sufficient substrates for charging tRNAs. To solve this problem, Zhang et al. introduced a threonine kinase PduX from *Salmonella* which is involved in coenzyme B_12_ biosynthesis into *E. coli* cells to produce a high level of intracellular pThr [109,110]. Secondly, because of the structural similarity between pThr and Sep, it is challenging to evolve SepRS to exclusively recognize pThr but not Sep. To overcome this issue, they used parallel positive selections combined with deep sequencing and statistical analysis to obtain mutually orthogonal SepRS variants to site-specifically incorporate pThr into recombinant proteins successfully [111].

## 9. Tyrosine Phosphorylation and its Analogs by Genetic Code Expansion

**Figure 7:**
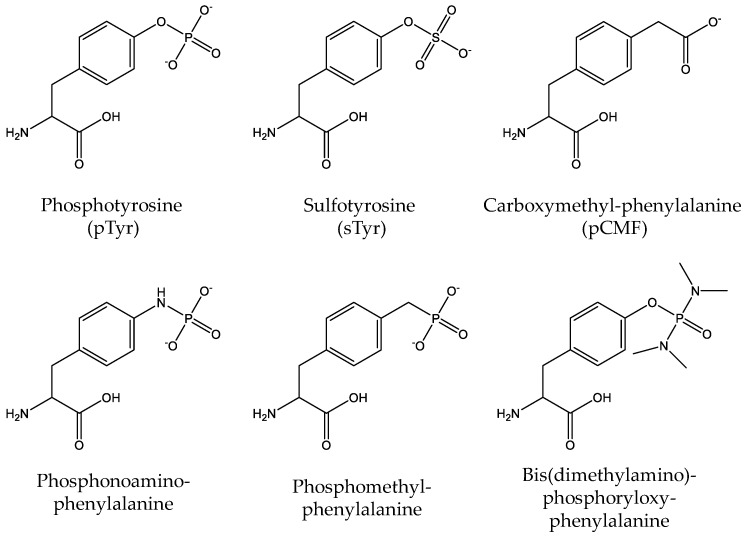
The structures of phosphotyrosine (pTyr) and its analogs, including sulfo-tyrosine (sTyr), *p*-carboxymethyl-phenylalanine (*p*CMF), *p*-(phosphonoamino)-phenylalanine, 4-phosphomethyl-phenylalanine and 3-4(bis(dimethylamino)phosphoryloxy) phenylalanine.

Different from Sep and pThr, there has been no known biosynthesis pathway in nature to generate phosphotyrosine (pTyr). The low permeability of pTyr through cell membranes makes it difficult to incorporate pTyr into proteins in living cells. Thus, Arslan et al. and Rothman et al. used cell-free translation systems to bypass such obstacles. By using chemically charged tRNAs with pTyr or its photocaged precursor, they successfully generated pTyr-containing proteins in vitro [112,113]. Later, several pTyr analogs were genetically incorporated into proteins in vivo (Figure 7). Liu et al. developed a genetic incorporation system for sulfo-tyrosine (sTyr), which is another PTM for tyrosine residues [114]. Due to the structure similarity, sTyr could be an ideal mimic of pTyr. Xie et al. genetically incorporated *p*-carboxymethyl-phenylalanine (*p*CMF) and demonstrated that *p*CMF could function as a pTyr mimic in the Y701 site of STAT1 [115]. Serwa et al. designed another pTyr analog, *p*-(phosphonoamino)-phenylalanine by chemically modifying a genetically installed *p*-azidophenyalanine residue [116].

Recently, three groups have independently developed genetic incorporation systems of pTyr in vivo. Fan et al. removed genes of five phosphatases from the *E. coli* genome to stabilize pTyr in cells. Combined with the *M. jannaschii* TyrRS (*Mj*TyrRS) variant and *E. coli* EF-Tu variant, which were screened for pTyr-tRNA formation and pTyr-tRNA binding respectively, we successfully incorporated pTyr into proteins in living *E. coli* cells [117]. Luo et al. used a different strategy to increase the cytoplasmic concentration of pTyr. They first synthesized a dipeptide (Lys-pTyr) which could be transported into cells by the dipeptide transporter DppA and then hydrolyzed into free Lys and pTyr by intracellular nonspecific peptidases. After solving the pTyr-uptake problem, they utilized an *Mj*TyrRS variant, which was previously engineered for *p*CMF incorporation to charge tRNA with pTyr and direct its incorporation into desired positions of proteins. By the same strategies, they also genetically incorporated a nonhydrolyzable analog of pTyr, 4-phosphomethyl-phenylalanine [118]. Meanwhile, Hoppmann et al. developed an efficient and easily accessible method to produce pure pTyr-containing proteins from a charge neutral and stable pTyr analog, 3-4(bis(dimethylamino)phosphoryloxy) phenylalanine, which can be converted into pTyr by pH shift [119].

## 10. Tyrosine Sulfation by Genetic Code Expansion

Tyrosine sulfation is a common and important PTM of membrane-bound and secretory proteins in eukaryotic cells [120,121]. It is essential to many cellular processes related to protein-protein interactions such as endogenous chemokine signaling (HIV-entry) [122]. To facilitate tyrosine sulfation studies, Liu et al. applied the genetic code expansion strategy to synthesize proteins with purely and site-specifically sulfated tyrosine residues [114]. In this study, they evolved the *Mj*TyrRS to incorporate sTyr into hirudin, which is used as an anticoagulant in medicine. Later, they optimized this system to allow the overexpression of objective proteins with site-specific tyrosine sulfation efficiently [123]. Recently, by introducing the C321.ΔA *E. coli* strain, Schwessinger et al. established a second-generation sTyr-incorporation system and demonstrated a practical application of this system in crop protection by synthesizing RaxX60-sY sulfated protein, which could activate immune response on rice with XA21 [124].

## 11. Tyrosine Nitration by Genetic Code Expansion

**Figure 8:**
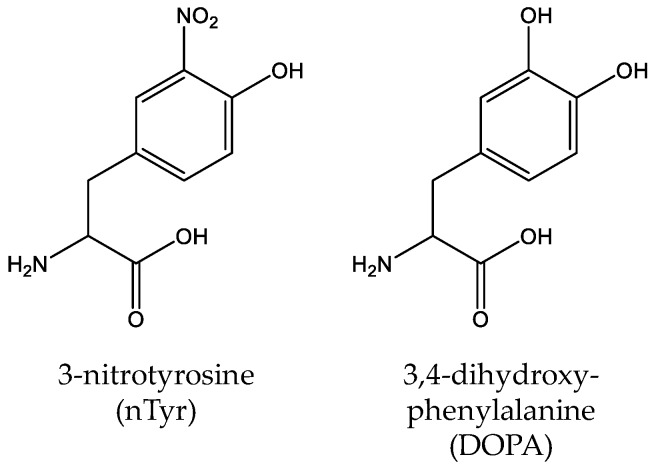
The structures of other modifications of tyrosine, including 3-nitro-tyrosine (nTyr) and 3,4-dihydroxy-phenylalanine (DOPA).

Unlike other PTMs, tyrosine nitration of proteins is a nonenzymatic process in which reactive nitrogen species such as peroxynitrite (ONOO^−^) and nitrogen dioxide (•NO2) initiate the oxidation of proteins and generate 3-nitro-tyrosine (nTyr) (Figure 8) [125]. Tyrosine nitration is involved in biological processes related to protein oxidation, causing many oxidative damage-related diseases including cancers and neurodegenerative disorders [126]. Taking Alzheimer’s disease (AD) as an example, a clinical study showed that the nTyr concentration of AD patients increased significantly (>6-fold) compared with controls [127]. To study the effects of tyrosine nitration, several genetic incorporation systems for nTyr have been established. Firstly, Neumann et al. evolved the *Mj*TyrRS to site-specifically incorporate nTyr into manganese superoxide dismutase (MnSOD) at position Tyr34 and showed that the nitration at this position could decrease > 97% enzyme activity of MnSOD alone [128]. Later, Cooley et al. used optimized selection protocols to engineer the *Mj*TyrRS to get an improved nTyr-incorporation system and successfully applied it into heat-shock protein studies to elucidate the role of elevated cellular nTyr levels in human disease [129,130]. By engineering the suppressor tRNA^Tyr^ with two substitutions in the anticodon loop (G34C/G37A), Rauch et al. further improved nTyr-incorporation efficiency [131]. Recently, Tack et al. utilized an engineered β-lactamase that is structurally dependent on nTyr-incorporation to study the evolving fitness of bacteria with an expanded genetic code and they found that after 2000 generations of directed evolution, the fitness deficit of cells related to nTyr toxicity was overcome by adaptive mutations [132]. The most commonly mutated or deleted genes were amino acid transporters in the hydroxyl and aromatic amino acid permease family such as the tyrosine-specific permease TyrP and the tryptophan permease Mtr. 

## 12. Tyrosine Hydroxylation by Genetic Code Expansion

3,4-dihydroxy-phenylalanine (DOPA) is a redox-active amino acid, which is naturally derived from tyrosine by co-translational or posttranslational modification (Figure 8) [133]. And it is involved in many biological processes such as the adhesive nature of the proteins [134]. In 2003, Alfonta et al. used an evolved *Mj*TyrRS variant to site-specifically incorporate DOPA into recombinant proteins in response to the amber codon UAG [135]. Later, Hauf et al. expanded the genetic code with a photocaged DOPA derivative (*o*-nitrobenzyl DOPA) through an engineered *Mj*TyrRS which could be cleaved by UV light [136]. Recently, Kim et al. constructed a bacterial strain which can biosynthesize DOPA by a tyrosine phenol-lyase and optimized the incorporation efficiency of DOPA [137]. Since DOPA can form a stable covalent linkage to the interacting protein, it has been used as a selective cross-linker which can detect the weak and/or transient protein interactions in protein complexes [138,139,140]. 

## 13. Summary and Perspective

For easy searching, we listed genetic incorporation systems for different PTMs in Table 1. It should be noticed that optimized systems may not always provide better incorporation efficiency, since ncAA incorporation depends on many factors including target protein properties, incorporation site contexts, expression strains and even growth media.

With the help of advance mass spectrometry techniques nowadays, detection of protein PTMs becomes much easier than ever before. And more and more studies have demonstrated that proteins usually have multiple PTMs which cross-interact with each other to modulate protein properties and functions [141]. This review focus on individual PTM incorporation but establishing a system for incorporating multiple PTMs into proteins simultaneously will be highly desirable. Recently, our group utilized mutually orthogonal SepRS and AcKRS systems in response to two stop codons to simultaneously incorporate Sep and AcK into target proteins. We also demonstrated mutual orthogonality of PylRS, *Mj*TyrRS and SepRS systems, implying the possibilities to incorporate three different PTMs into a single protein [142]. Similarly, Wright et al. produced site-specifically acetylated Thioredoxin reductase 1 that also contains selenocysteine (Sec) by simultaneous UAG codon reassignment to AcK and UGA codon recoding to Sec [143]. They also demonstrated another strategy to produce dual-modified proteins, combining the Sep-incorporation system and enzymatic phosphorylation to synthesize the proto-oncogene Akt protein with dual-phosphorylation at S473 and T308 simultaneously [144].

Besides PTMs listed above, there are many other PTMs in nature such as phosphorylation of aspartic acid and histidine [145], carboxylation of glutamic acid [146], carbohydration of asparagine [147], which are also play important roles in biological processes. Developing genetic incorporation systems for them is another major direction for this field. However, due to the distinct structures from substrates of PylRS, TyrRS and SepRS, commonly-used orthogonal pairs may not be proper for those PTMs. So *de novo* engineering of new orthogonal pairs from other AARSs should be necessary.

## Figures and Tables

**Table 1:** An index of genetic incorporation systems for PTM studies.

PTM Types	Noncanonical Amino Acids (AARSs Derived From)	References
*Lysine acetylation*		
	Acetyllysine (PylRS)	32,34,38
Analog	Thio-acetyllysine (PylRS)	46
	2-Amino-8-oxononanoic acid (PylRS)	44
	Trifluoro-acetyllysine (PylRS)	47
*Lysine ubiquitination*		
Precursor	d-Cys-ε-Lys (PylRS)	63
	δ-Thiol-lysine (PylRS)	64
*Lysine methylation*		
Mono-methylation precursor	Boc-methyllysine (PylRS)	73
	*o*-Nitrobenzylcarbamoyl-methyllysine (PylRS)	74,75
	N^ε^-allylcarbamoyl-methyllysine (PylRS)	76
Di-methylation precursor	Boc-methyllysine (PylRS)	78
	*N*^ɛ^-(4-azidobenzoxycarbonyl)-δ, ɛ-dehydrolysine (PylRS)	79
Tri-methylation precursor	Phosphoserine (SepRS)	80
*Other lysine acylation*		
	Crotonyllysine (PylRS)	84,85,87
	Propionyllysine (PylRS)	85
	Butyryllysine (PylRS)	85,87
	2-Hydroxyisobutyryllysine (PylRS)	86
Precursor	Azidonorleucine (PylRS)	88
*Arginine methylation*		
	Monomethyl-arginine (yeast ArgRS)	91
*Serine phosphorylation*		
	Phosphoserine (SepRS)	100,101,106
Analog	Phosphonomethylene alanine (SepRS)	108
*Threonine phosphorylation*		
	Phosphothreonine (SepRS)	112
*Tyrosine phosphorylation*		
	Phosphotyrosine (*Mj*TyrRS)	118–120
Analog	Carboxymethyl-phenylalanine (*Mj*TyrRS)	116
	4-Phosphonomethyl-phenylalanine (*Mj*TyrRS)	119
	*p*-(Phosphonoamino)-phenylalanine (*Mj*TyrRS)	117
	Sulfotyrosine (*Mj*TyrRS)	115
*Tyrosine sulfation*		
	Sulfotyrosine (*Mj*TyrRS)	115,124,125
*Tyrosine nitration*		
	3-Nitro-tyrosine (*Mj*TyrRS)	129–133
*Tyrosine hydroxylation*		
	3,4-Dihydroxy-phenylalanine (*Mj*TyrRS)	136–138
